# The agreement of the various distance walkway in the 6-minute walk test in healthy adults

**DOI:** 10.1371/journal.pone.0321503

**Published:** 2025-04-28

**Authors:** Busaba Chuatrakoon, Natakit Seepang, Derin Chaiwong, Rawes Nanavichit, Kittipan Rerkasem, Sothida Nantakool

**Affiliations:** 1 Department of Physical Therapy, Integrated Neuro-Musculoskeletal, Chronic Disease, and Aging Research Engagement Center (I-CARE center), Faculty of Associated Medical Sciences, Chiang Mai University, Chiang Mai, Thailand; 2 Environmental-Occupational Health Sciences and Non Communicable Diseases Research Center, Research Institute for Health Sciences, Chiang Mai University, Chiang Mai, Thailand; 3 Department of Surgery, Faculty of Medicine, Chiang Mai University, Chiang Mai, Thailand; 4 Faculty of Medicine, Clinical Surgical Research Center, Chiang Mai University, Chiang Mai, Thailand; Universidade de Aveiro Escola Superior de Saude de Aveiro, PORTUGAL

## Abstract

**Background:**

Despite a practical guideline of 30-meter walking path during 6-minute walk test (6MWT), such walking course length is not possible in every clinical setting due to unavailable sufficient space. Existing evidence has investigated using several shorter course lengths, it remains unclear whether a walking course length shorter than the standard walking course length is appropriate for 6MWD testing. This study aimed (i) to compare maximum walking distances at various shorter walking course lengths (i.e., 10, 20, and 25 meters) and 30 meters, and (ii) to assess agreements in maximum walking distances achieved at intervals below 30 meters, specifically 10, 15, 20, and 25 meters.

**Methods:**

This study was a cross-sectional with cross-over design. Forty-eight healthy participants were randomly ordered to perform 6MWT with five different walkways (10, 15, 20, 25, 30 meters). The maximum walking distance (six-minute walk distance, 6MWD) covered was recorded.

**Results:**

Eligible participants aged 41.0 ± 17.2 years, with equal sex (24 males) participated in this study. The 6MWD at 10, 15, and 20-meter walkways significantly shorter than the 30-meter standard walkway (489.6 ± 59.3 m, 513.1 ± 62.6 m, 524.7 ± 63.7 m vs 539.1 ± 63.1 m, respectively (P<0.01)). Very strong agreement was observed at 15, 20, and 25 meters with the standard 30 meters (0.819–0.875, P<0.001). Subgroup analysis showed strong to very strong agreement in 10-meter walkway length onwards with the standard walkway length among older adults (0.757–0.918, P<0.001).

**Conclusions:**

Testing on 20 meters walkway and shorter yielded varied results compared to the standard 30-meter walk, with exceptional congruence observed at 15 meters onwards. In particular, a minimum walkway of 10 meters had strong agreement with a standard 30-meter walkway in elderly.

## Introduction

Physical performance serves as a crucial indicator of the body’s efficiency in daily activities and its ability to promptly return to a normal state, significantly contributing to overall health [1]. This multifaceted concept encompasses muscle flexibility, strength, endurance, as well as respiratory and cardiovascular fitness. Evaluating physical performance is pivotal for understanding an individual’s capacity to lead a healthy life, with various tests designed for specific assessment objectives [2].

Functional assessments, which offer insights into overall health during activities, involve evaluating multiple body systems, including respiratory, cardiovascular, and muscular systems [2,3]. While laboratory-based assessments in controlled settings, such as the cardiopulmonary exercise test (CPET), treadmill test, and cycling ergometer test, provide important physiological parameters, such as maximum oxygen consumption (VO_2max_) and energy expenditure, they often necessitate expensive equipment and require expertise. To address this, more accessible field-based assessments have been warranted [4].

The 6-minute walk test (6MWT), introduced by Balke in 1963, is a widely accepted field-based assessment for physical performance [5]. With respect to the test, various internal and external factors affecting the 6MWT such as age, gender, height, body weight, comorbidities, and walking course length [6–8]. The American Thoracic Society has suggested a practical guideline using a 30-meter walking path as a standard walking course length during 6MWT [6]. However, such walking course length is not possible in every clinical setting due to unavailable sufficient space. Consequently, a shorter course length is needed. A growing body of evidence has debatable effects of different walking course length in individuals with various ages and disease conditions [9–11]. While some previous studies found a comparable 6MWD between 15- and 30-meter walking course length [9], some other studies showed a different walking distance between 15- and 30-meter walking course length [10, 11]. It remains unclear whether a walking course length shorter than the standard walking course length is appropriate for 6MWD testing. Limited walkway length in clinical environments poses a significant challenge for accurately assessing functional performance using the 6MWT. Addressing this gap can enhance the feasibility and accuracy of the test, thereby improving patient care and outcomes. Therefore, this study aims to evaluate shorter walkway lengths as viable alternatives, which could facilitate broader implementation of the 6MWT in space-constrained settings, ultimately benefiting clinical practice by providing reliable and accessible functional performance assessments.

## Material and methods

### Ethics approval

All participants provided informed consent before participating in the study. This study was approved by the Research Ethics Committee, Faculty of Associated Medical Sciences, Chiang Mai University (AMSEC-66EX-053).

### Study design and participants

This study was cross-sectional and cross-over design, investigating walking distances at various lengths—specifically, 10, 15, 20, and 25 meters—compared to the standard 30-meter walking length during a 6MWT in 48 healthy participants. Participants were recruited from 1 December 2023–28 February 2024 via a public advertisement. Inclusion criteria were (i) participants aged between 20 and 70 years, (ii) both genders, and (iii) stable health condition. Any participants who (i) were unable to walk independently or using walking aid, (ii) had balance impairment, (iii) had uncorrected vision problems, (iv) had middle ear-related disorders affecting posture, (v) had chronic respiratory or circulatory conditions, or (vi) musculoskeletal disorders influencing walking tests were excluded.

### Sample size calculation

Due to data limitations from similar previous studies, the sample size estimation is based on a cross-over design using G*Power software [12]. The statistical tests include F-test, Repeated Measures, within factors, utilizing the following statistical parameters. For the sample size calculation, the following parameters were used effect size (medium) = 0.2, alpha = 0.05, power = 0.95, number of groups = 1 and number of measurements = 5. With these parameters, the calculated sample size is 48 individuals.

### Procedure and outcomes

**Fig 1:**
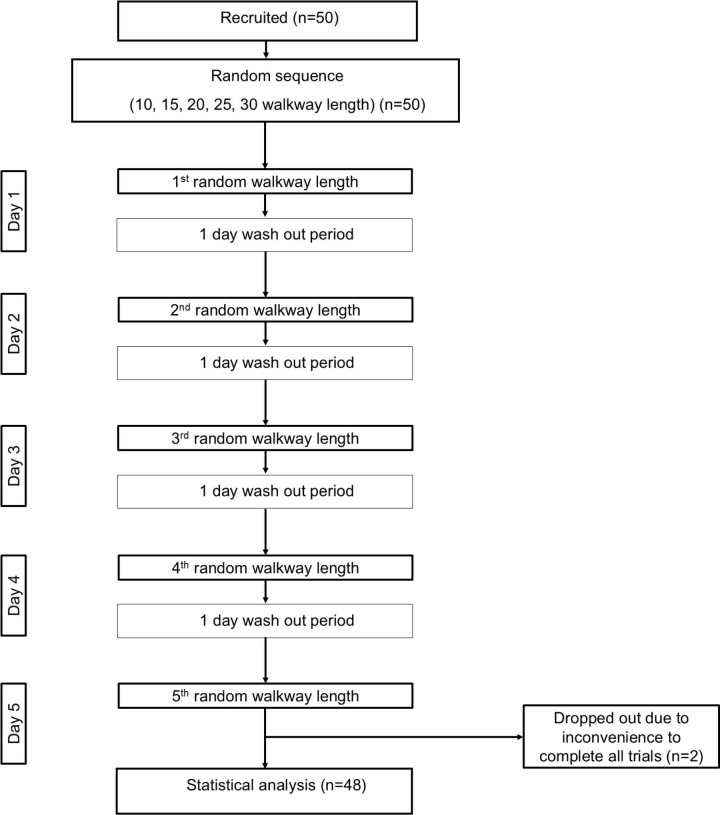
Flowchart of study procedure.

Eligible participants were randomly assigned the sequence of 6MWT at 10, 15, 20, 25, and 30 meters. The five tests were assigned in a random order using a computer-generated sequence during the first visit of each participant to ensure transparency and minimize bias. Each participant underwent 6MWT once per day, with a one-day interval to prevent fatigue. The participants walked the designated distance under each condition (Fig 1). Testing was conducted following the standard guidelines [6]. Participants were asked to walk on a long-flat corridor at their self-selected pace for a total time of six minutes. Participants were provided with an opportunity to perform a practice trial on the enrollment day, distinct from the data collection period, to ensure familiarity with the procedure and confirm their ability to complete the test. The test was conducted twice, with a minimum of 30 minutes rest between the tests. Vital signs (i.e., heart rate, oxygen saturation, and blood pressure), rating perceived exertion using Borg scale, and other adverse events were monitored during testing. 6MWT was terminated if participants had adverse symptoms—such as palpitations, pallor, chest pain, or shortness of breath—prevent continued walking. The 6MWD achieved during the 6MWT at different walking course lengths was recorded. A well-trained assessor (single assessor) conducted all tests, following the standard instructions of the American Thoracic Society (ATS), 2002, to ensure consistency and replicability [6].

### Statistical analysis

Descriptive statistics were used to describe demographic data. The Shapiro wilk test was used to detect normality of data distribution. Differences in maximum walking distance, heart rate, and rating perceived exertion during walks (1 group x 5 conditions) using one-way repeated analysis of variance (ANOVA), with Bonferroni correction. Agreements of 6MWD at 10-, 15-, 20-, 25-, and 30-meters walking course length (6MWD-10, 6MWD-15, 6MWD-20, 6MWD-25 and 6MWD-30, respectively) were calculated using the Lin concordance between different walking course lengths. Subgroup analysis of age (i.e., 20–39, 40–59, and ≥60 years) was also analysed. According to Altman’s suggestion, the agreement is interpreted as very weak (0.0–0.19), weak (0.2–0.39), moderate (0.4–0.59), strong (0.6–0.79), and very strong (0.8–1.0) [13]. A significance level was set at p < 0.05.

## Results

The demographic data of participants were reported in Table 1.

**Table 1:** Demographic data of participants.

Variables	N = 48 (mean ± SD)
Age (y)	41.0 ± 17.2
Sex (M, %)	24 (50)
Weight (kg)	63.0 ± 13.7
Height (cm)	162.6 ± 0.1
BMI (kg/m^2^)	23.7 ± 4.3

cm, centimeter; kg, kilogram; kg/m^2^, kilogram per square meter; M, male; SD, standard deviation; y, year.

**Fig 2:**
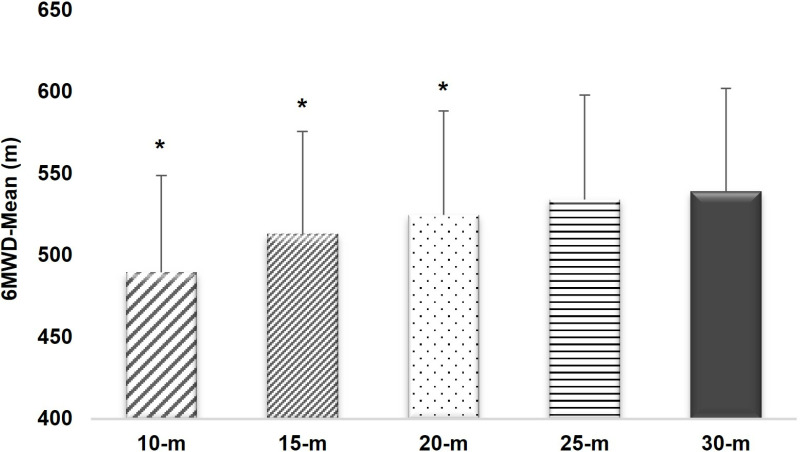
Comparison of 6MWD among various walking course lengths. *, p-value < 0.05 vs 30-m.

The comparison of 6MWD in various walking course lengths was described in Table 2 and Fig 2. No differences were observed in 6MWD-30 and 6MWD-25, while significant differences were found in 6MWD-10, 6MWD-15, and 6MWD-20 when compared to the standard walking course length. There were no differences in vital signs and Borg scale ratings after each condition test.

**Table 2:** Comparison of 6MWD among various walkway lengths.

Outcomes	Mean ± SD	F	p-value	Partial Eta Squared
6MWD-10	489.6 ± 59.3	F4,188=38.248 P=<0.001	<0.001*	0.449
6MWD-15	513.1 ± 62.6		<0.001*	
6MWD-20	524.7 ± 63.7		0.01*	
6MWD-25	534.3 ± 63.8		1.00	
6MWD-30	539.1 ± 63.1		Ref.	

6MWD, six-minute walk distance; SD, standard deviation; *, p-value < 0.05.

The agreement of 6MWD in various walking course lengths was reported in Table 3. The agreement of 6MWD in all walking course lengths was significantly strong to very strong associated with the standard 30-meter walking course length.

**Table 3:** Agreement of 6MWD among different walking course lengths.

Outcomes	Mean ± SD	Lin concordance correlation coefficient (ρc)	95% CI	p-value
6MWD-10	489.6 ± 59.3	0.669	0.555-0.782	<0.001
6MWD-15	513.1 ± 62.6	0.819	0.733-0.905	<0.001
6MWD-20	524.7 ± 63.7	0.875	0.809-0.941	<0.001
6MWD-25	534.3 ± 63.8	0.862	0.787-0.936	<0.001
6MWD-30	539.1 ± 63.1	Ref.	Ref.	Ref.

6MWD, six-minute walk distance; SD, standard deviation.

Subgroup analysis by age found that, in age groups of 20–39 and 40–59 years, there were significantly strong to very strong agreements between 15-, 20-, 25-meter and the standard walking course lengths, except for 10-meter walking course length which had a significantly moderate agreement with the standard walking course lengths. In the age group of ≥60 years, there were significantly strong to very strong agreements of all walking course lengths and the standard walking course length (Table 4).

**Table 4:** Agreement of 6MWD among different walking course lengths in subgroup analyses of age.

Outcomes	Age range (n)	Mean ± SD	Lin concordance correlation coefficient (ρc)	95% CI	p-value
6MWD-10	20-39 (n=20)	530.3 ± 42.0	0.481	0.284-0.678	<0.001
6MWD-15		552.7 ± 52.6	0.684	0.488-0.880	<0.001
6MWD-20		569.4 ± 57.1	0.823	0.689-0.956	<0.001
6MWD-25		576.2 ± 57.1	0.750	0.560-0.940	<0.001
6MWD-30		584.5 ± 46.5	Ref.	Ref.	Ref.
6MWD-10	40-59 (n=19)	466.8 ± 44.5	0.481	0.253-0.709	<0.001
6MWD-15		489.5 ± 52.8	0.747	0.571-0.923	<0.001
6MWD-20		499.7 ± 39.7	0.727	0.536-0.918	<0.001
6MWD-25		511.9 ± 39.2	0.723	0.519-0.926	<0.001
6MWD-30		516.4 ± 51.2	Ref.	Ref.	Ref.
6MWD-10	≥60 (n=9)	445.0 ± 70.4	0.757	0.523-0.991	<0.001
6MWD-15		473.3 ± 57.8	0.827	0.581-1.072	<0.001
6MWD-20		475.1 ± 61.5	0.866	0.679-1.053	<0.001
6MWD-25		485.6 ± 72.1	0.918	0.843-0.994	<0.001
6MWD-30		482.0 ± 52.0	Ref.	Ref.	Ref.

6MWD, six-minute walk distance; SD, standard deviation.

## Discussion

This study demonstrated that 6MWD results from 15-meter walkways and above strongly agree with the standard 30-meter walkway, with age-related differences in agreement patterns. Despite a standard walkway length for 6MWT, functional performance assessed by 6MWT is limited due to insufficient walkway length test. Several previous studies have investigated a shorter walkway length which is expected as an alternative walkway length [9–11,14]. Our study reveals a reduction in walking distances during the 6MWT as the path length decreases. Significant differences between 20-meter path length and lower, and the standard 30-meter walk were consistent with the previous evidence which investigated walking distances in healthy individuals aged 50 years and older during 6MWTs with path lengths of 10, 20, and 30 meters [14]. The results found that covered distances on 20- and 10-meter walkway were 18 and 59 meter lower than the standard 30-meter walkway, respectively [14]. Greater walking distances in longer walkway lengths may be attributable to more space for acceleration, higher maximum speeds, and influencing the number of turns [14]. Our findings also in line with studies in individuals with COPD, demonstrating that the 6MWD in 10- and 20-meter had more turns than those with a 30-meter walkway resulting in the shorter distance in 10- and 20-meter walkway than 30-meter walkway [10,15]. The increased turning frequency required on shorter walkways likely contributes to greater energy expenditure and impacts overall walking distances. This factor is crucial to consider when evaluating the feasibility and accuracy of shorter walkways for the 6MWT.

Although there were differences in walking distances observed in tests with path lengths 20 meters and shorter, the current study highlights that the walking distances obtained at a path length of 15 meters onwards align remarkably well with the distances achieved in the standard 30-meter walk. This finding suggests that a 15-meter walkway may be used as a minimum alternative walking course length during 6MWT. The subgroup analysis based on three different age ranges (20–39, 40–59, ≥60 years) provides valuable insights into the functional performance of specific population groups. Evaluating the 6MWT results across these age categories helps to understand how age-related factors influence walking distance and overall functional capacity. This information is crucial for tailoring clinical interventions and rehabilitation programs to meet the unique needs of different age groups. For instance, recognizing that older adults may have different functional performance benchmarks can assist clinicians in setting more realistic and achievable goals for this population, ultimately enhancing patient outcomes. Subgroup analysis by age ranges found that young adults demonstrated outstanding congruence when tested at a 20-meter walkway onwards, while middle-aged adults exhibited remarkable congruence from the 15-meter interval onwards. Older adults demonstrated consistent congruence at all test intervals. These findings suggest that the impact of walkway length on test outcomes diminishes as age increases, possibly due to the slower walking speeds inherent in the elderly. It is likely that deceleration during the turning phase does not affect the overall walking distance in the tests among older adults. This finding contradicts previous evidence reporting that older adults experienced slowing down, resulting in a decreased walking distance when tested on walkways shorter than 20 meters [16]. The strong agreement observed at 10-meter walkways in older adult contrasts with the previous study suggesting performance declines with shorter walkways. This discrepancy may be attributed to differences in the testing protocols used to evaluate gait speed and the testing environment.

This study has certain limitations that should be considered. A relatively small number of participants when categorized by age, may limit the generalizability of the results. The findings observed in healthy individuals may not necessarily translate to populations with specific health conditions, such as respiratory or cardiovascular diseases. This limitation underscores the need for further research to assess the agreement between different walkway lengths in such populations. Nevertheless, the overall conclusion drawn from the study is that testing at a walkway length equal to and shorter than 20 meters yields different outcomes compared to the standard 30-meter walk, and exceptional congruence is observed when testing at walkway lengths from 15 meters and above. These findings are particularly important for space-constrained settings, such as small clinics or rehabilitation centers, where the ability to use shorter walkways for the 6MWT can significantly enhance the feasibility of conducting this test. This ensures that accurate functional performance assessments can be made even when space is limited, ultimately improving patient care and outcomes in these environments.

## Conclusion

This study concludes that 20 meters walkway and shorter yielded varied results compared to the standard 30-meter walk. A minimum walkway of 15 meters had very strong agreement with a standard 30-meter walkway. In particular, a minimum walkway of 10 meters had strong agreement with a standard 30-meter walkway in elderly. These findings provide practical guidance for implementing the 6MWT in clinical settings where a standard 30-meter walkway is unavailable, improving accessibility without compromising test validity.
